# Linalool and Geraniol Defend Neurons from Oxidative Stress, Inflammation, and Iron Accumulation in In Vitro Parkinson’s Models

**DOI:** 10.3390/antiox13080917

**Published:** 2024-07-29

**Authors:** Edina Pandur, Balázs Major, Tibor Rák, Katalin Sipos, Adrienne Csutak, Györgyi Horváth

**Affiliations:** 1 Department of Pharmaceutical Biology, Faculty of Pharmacy, University of Pécs, 7624 Pécs, Hungary; edina.pandur@aok.pte.hu (E.P.); majbazsa@gmail.com (B.M.); katalin.sipos@aok.pte.hu (K.S.); 2 Department of Ophthalmology, Medical School—Clinical Centre, University of Pécs, 7624 Pécs, Hungary; rak.tibor@pte.hu (T.R.); csutak.adrienne@pte.hu (A.C.); 3 Department of Pharmacognosy, Faculty of Pharmacy, University of Pécs, 7624 Pécs, Hungary

**Keywords:** linalool, geraniol, Parkinson’s disease, inflammation, iron, ROS, antioxidant

## Abstract

Parkinson’s disease is one of the most prevalent neurological disorders affecting millions of people worldwide. There is a growing demand for novel and natural substances as complementary therapies. Essential oils and their various compounds are highly investigated natural plant-based products as potential treatment options for common human diseases, such as microbial infections, chronic diseases, and neurodegenerative disorders. The present study focuses on the beneficial effects of linalool and geraniol, the major compounds of lavender (*Lavandula angustifolia* L.) and geranium (*Pelargonium graveolens* L’Hér. in Aiton) essential oils, on oxidative stress, inflammation, and iron metabolism of the rotenone and 6-hydroxydopamine-induced in vitro Parkinson’s models. The experiments were carried out on all-trans retinoic acid differentiated SH-SY5Y cells. The effects of linalool and geraniol were compared to rasagiline, an MAO-B inhibitor. The results revealed that both essential oil compounds reduce the level of reactive oxygen species and alter the antioxidant capacity of the cells. They lower the secretion of IL-6, IL-8, and IL-1β pro-inflammatory cytokines. Moreover, linalool and geraniol change the expression of iron-related genes, such as the iron importer transferrin receptor 1, heme-oxygenase-1, and ferroportin iron exporter, and influence the intracellular iron contents. In addition, it has been unveiled that iron availability is concatenated with the actions of the essential oil compounds. Based on the results, linalool and geraniol are vigorous candidates as an alternative therapy for Parkinson’s disease.

## 1. Introduction

Neurodegenerative disorders are emerging health problems worldwide [1]. Parkinson’s disease is one of the most prevalent neurodegenerative diseases, affecting around 10 million people worldwide [2]. Parkinson’s disease causes motor and non-motor dysfunctions, such as tremors, rigidity, bradykinesia, insomnia, and depression [3].

Several molecular mechanisms, including neurotrophic factor deprivation, or the inhibition of inflammatory cascade, oxidative stress, mitochondrial dysfunction, glutamate-excitotoxicity, autophagy dysregulation, protein misfolding, ischemia, and hypoxia, may contribute to the etiology and progression of neurodegenerative diseases [4]. Parkinson’s disease induces the synthesis of α-synuclein and the formation of Lewy bodies in dopaminergic neurons, leading to oxidative stress, mitochondrial dysfunction, decreased dopamine production, and neuronal cell death [5]. Neuroinflammation can contribute to neurodegeneration by the overactivation of microglial cells and the excess release of pro-inflammatory cytokines [5,6]. Fractalkine, produced by the neurons, acts as the regulator of microglial activity, is also implicated in neuroinflammation, and contributes to the progression of the disease [7].

Despite typical motor manifestations, Parkinson’s can also involve the visual pathway, causing reduced contrast sensitivity, motion perception abnormalities, and color deficiencies [8]. Zhao et al. presented the hypothesis that glaucoma might be a non-motoric precursor symptom to the onset of Parkinson’s disease [9].

Parkinson’s disease causes the deterioration of the central nervous system by affecting the dopaminergic neurons of the substantia nigra, which are the regulators of motor functions, and also affects the interactions between the brain and spinal cord [10]. Dopaminergic amacrine cells have also been found in the human retina, whose cell bodies are located in the inner nuclear layer of the retina, connecting photoreceptors and ganglion cells [8].

It has been revealed that dysregulation of iron metabolism and iron accumulation in the substantia nigra is associated with oxidative damage and the loss of dopaminergic neurons [11,12]. Ferroptosis is also involved in Parkinson’s disease and pathologically high intraocular pressure-induced glaucomatous retinal ganglion cell loss, closely regulated by iron metabolism [13].

The treatment of Parkinson’s disease targets α-synuclein to reduce aggregation and dopamine degradation by utilizing monoamine oxidase B inhibitors (MAO-B), neuroinflammation, and iron accumulation to decrease oxidative stress and neuronal cell death [14,15,16,17]. New therapeutic approaches are still being investigated to improve the patient’s prospects.

Essential oils are mostly pleasant-scented liquids that mainly contain a mixture of mono- and sesquiterpenes and phenylpropane derivatives [18]. Essential oils are widely used natural compounds in treating many diseases (e.g., bacterial and viral infections, chronic diseases, and inflammatory diseases) [18,19,20]. Essential oils are considered to be good options for neuroprotective treatments for many neurodegenerative illnesses because of their anti-inflammatory and antioxidant characteristics as well as their ability to permeate across the blood–brain barrier [21,22,23]. *Cinnamomum verum* J. Presl, *Rosmarinus officinalis* L., *Lavandula angustifolia* Mill., *Citrus* × *sinensis* (L.) Osbeck, *Mentha* × *piperita* L., *Thymus vulgaris* L., *Zingiber officinale* Roscoe, and *Rosa damascena* Mill. have been examined in different in vitro and in vivo Parkinson’s models and were proven to have a beneficial effect on oxidative stress, inflammation, α-synuclein aggregation, and apoptosis [24,25,26,27].

According to the literature, *Lavandula angustifolia* L. essential oil inhalation alleviates inflammation by decreasing the production of pro-inflammatory cytokines and reducing depression [28]. Linalool, the main compound in lavender oil, has been shown to reduce inflammation in macrophages [29]. Linalool attenuates the loss of dopaminergic neurons and improves motor function in mice treated with 1-methyl-4-phenyl-1,2,3,6-tetrahydropyridine (MPTP) [30]. Geraniol possesses antimicrobial and anti-cancer activities [31] and was shown to ameliorate memory functions and synaptic plasticity in a rat model of Alzheimer’s disease [32].

In our study, we examined the effects of linalool, the major component of *Lavandula angustifolia* Mill essential oil, and geraniol, the main compound of *Pelargonium graveolens* L’Hér. in Aiton essential oil, in rotenone or 6-hydroxydopamine (6-OHDA)-induced Parkinson’s cell culture models. The results revealed that both essential oil compounds reduce oxidative stress by modifying the antioxidant capacity of the differentiated SH-SY5Y cells. They lower the pro-inflammatory cytokine secretion. Moreover, linalool and geraniol alter the expression of iron-related genes and the iron accumulation in the cells, which has not been investigated yet. Based on the results, linalool and geraniol are potent candidates as an alternative therapy for Parkinson’s disease.

## 2. Materials and Methods

### 2.1. Cell Cultures and Treatments

The SH-SY5Y neuroblastoma cells (ATCC, CRL-2266) were cultured in Dulbecco’s Modified Eagle Medium/Nutrient Mixture F-12 medium (DMEM/F12; Capricorn Scientific GmbH, Ebsdorfergrund, Germany) supplemented with 10% fetal bovine serum (FBS, Capricorn Scientific GmbH, Ebsdorfergrund, Germany) and 1% non-essential amino acids (NEAA, BioWest, Nuaillé, France) and 1% penicillin–streptomycin (P/S, BioWest, Nuaillé, France). The cells were differentiated into dopaminergic neurons with 1 μM all-trans retinoic acid (ATRA, Merck Life Sciences Kft, Budapest, Hungary) for 5 days using serum-depleted (1% FBS) culture medium. In the first experiment, the cells were treated with 5 μM rotenone (Merck Life Sciences Kft, Budapest, Hungary) or 150 μM 6-hydroxidopamine hydrobromide (6-OHDA, Merck Life Sciences Kft, Budapest, Hungary) for 24 h to generate the chemical background of Parkinson’s disease. After developing the Parkinson’s model, the cells were treated with (−)-linalool (Merck Life Sciences Kft, Budapest, Hungary) using a 1000-fold dilution of the stock solution (0.54 μg/mL) or geraniol (Merck Life Sciences Kft, Budapest, Hungary) using a 500-fold dilution of the stock solution (0.84 μg/mL). For positive control of the experiments, 1 μM of rasagiline mesylate (Merck Life Sciences Kft, Budapest, Hungary), an anti-Parkinson’s drug (monoamine oxidase B inhibitor), was used. Each treatment lasted 24 h. In the second experiment, the cells were treated with 100 μM ferric ammonium citrate (FAC, Merck Life Sciences Kft, Budapest, Hungary) and rotenone or 6-OHDA together for 24 h to generate increased iron uptake of the cells, a characteristic of Parkinsonian neurons. Then, the cells were treated the same way as in the first experiment. In the third experiment, the iron accumulation of the neurons was modeled using 24 h long FAC pretreatment. Then, the cells were treated the same way as in the first experiment setting. The appropriate treatment concentrations were determined separately for each agent using resazurin-based cell viability assays (Toxo-8 kit, Merck Life Technologies Kft, Budapest, Hungary). The viability results are shown in Supplementary Figure S1. Stock solutions of linalool and geraniol standards were prepared freshly by adding 10% of 100% dimethyl sulfoxide (100 μL DMSO, Merck Life Sciences Kft, Budapest, Hungary) to 90% of essential oil component (900 μL). The emulsions were mixed by rigorous vortexing and then were diluted with the cell culture medium. The control cells, the only FAC-treated cells, the only rotenone, and the only 6-OHDA-treated cells were supplemented with an equal amount of DMSO, as in the linalool and geraniol treatments. All experiments were conducted in a humidified atmosphere containing 5% CO_2_ at 37 °C.

### 2.2. Determination of the Reactive Oxygen Species (ROS) Production

The cells were seeded onto a 96-well plate with 10^4^ cell density/well. The cells were differentiated and treated according to the three experimental protocols. The ROS production of the living cells was determined using the Fluorometric Intracellular ROS Kit (Merck Life Technologies Kft, Budapest, Hungary). After adding the ROS detection reagent, the cells were incubated for 30 min. The fluorescence intensity was measured at λex 640/λem 675 nm wavelength using an EnSpire Multimode microplate reader (PerkinElmer, Rodgau, Germany) with a bottom read mode. The ROS level was expressed in the percentage of the control cells, which was considered 100%.

### 2.3. Total Antioxidant Capacity (TAC) Measurements

The cells were cultured in 6-well plates at a 5 × 10^5^ cell density/well. After differentiation, the cells were treated according to the protocols described in Section 2.1. The cells were collected by trypsinization and pelleted by centrifugation. The cell pellets were lysed in cold phosphate-buffered saline (PBS). The concentration of both small-molecule and protein antioxidants was determined using the Total Antioxidant Capacity Assay Kit (Merck Life Sciences Kft, Budapest, Hungary). The concentration of the antioxidants was expressed as Trolox equivalents in nmol/μL.

### 2.4. Enzyme-Linked Immunosorbent Assay (ELISA)

After each treatment of the cells, the culture media of the control and treated cells were collected and stored at −80 °C until the measurements. The IL-6, IL-1β, IL-8, and fractalkine secretions of the cells were determined by human IL-6, human IL-8, human IL-1β, and human fractalkine ELISA kits (Thermo Fisher Scientific Inc., Waltham, MA, USA) according to the manufacturer’s protocols.

### 2.5. Intracellular Iron Content Determination

The intracellular iron content was determined using a colorimetric ferrozine-based assay. The cells were cultured and treated in 6-well plates. The neurons were collected by centrifugation and lysed with 200 μL of 50 mM NaOH at room temperature for 2 h. After the incubation, 100 μL of the samples was mixed with 100 μL of iron-releasing reagent (1.4 M HCl, 4.5% (*w*/*v*)) and was incubated for 2 h at 60 °C. After iron was released from proteins, 30 μL of iron detection reagent (6.5 mM ferrozine, 6.5 mM neocuproine, 2.5 M ammonium acetate, 1 M ascorbic acid) was added to the samples and incubated at room temperature for 30 min. The absorbance was measured in 96-well dishes at 550 nm using a Multiskan GO spectrophotometer (Thermo Fisher Scientific Inc., Waltham, MA, USA). The concentration was determined by a standard curve of FeCl3 treated the same way as the samples. For the normalization of the iron measurements, the protein concentration was determined from each sample with the DC Protein Assay Kit (Bio-Rad Inc., Hercules, CA, USA). The intracellular iron content was expressed as μM iron/mg protein.

### 2.6. ATP Measurements

The cells were cultured in 6-well plates at a 5 × 10^5^ cell density/well. After differentiation, the cells were treated according to the protocols. The cells were collected by trypsinization and pelleted by centrifugation. The cells were used immediately for ATP determination. The ATP concentration of the samples was measured using the ATP Colorimetric/Fluorometric Assay Kit (Merck Life Sciences Kft, Budapest, Hungary), and we used the colorimetric protocol at 570 nm wavelength. The results were expressed as ng/μL.

### 2.7. Real-Time PCR

After the treatments, the differentiated SH-SY5Y cells were washed with phosphate-buffered saline (PBS; Capricorn Scientific GmbH, Ebsdorfergrund, Germany) and harvested by trypsinization. Total RNA isolation was performed using the Aurum Total RNA Isolation Kit (Bio-Rad Inc., Hercules, CA, USA). Complementary DNA was synthesized from 200 ng total RNA using iScript cDNA Kit (Bio-Rad Inc., Hercules, CA, USA) according to the manufacturer’s protocol. Real-time PCR was performed in the Opus 96 Real-Time System (Bio-Rad Inc., Hercules, CA, USA) with iTaq™ Universal SYBR^®^ Green Supermix (Bio-Rad Inc., Hercules, CA, USA). The total reaction volume was 20 μL. The amplification specificity was verified by generating melting curves after each qPCR run. The relative gene expression was calculated using the Bio-Rad CFX Manager 3.1 software (Bio-Rad Inc., Hercules, CA, USA). For normalization, the glyceraldehyde-3-phosphate dehydrogenase was applied. The relative expression rates were compared between the treated and the control samples. The relative expression of the controls was considered as 1. The primer sequences used in this study are described in Table 1.

### 2.8. Data Analysis

The data presented are representative of three independent experiments. The number of technical replicates was four for viability and ROS measurements and three for all additional experiments. Statistical analysis was performed using SPSS software version 24.0 (IBM Corporation, Armonk, NY, USA). The normality of the data was analyzed with the Shapiro–Wilk test, and after verifying its distribution, the statistical significance was determined using one-way ANOVA followed by Tukey’s post hoc test. Data are shown as mean ± standard deviation. Statistical significance was set at *p*-value < 0.05.

## 3. Results

### 3.1. Linalool and Geraniol Suppress Reactive Oxygen Species Generation

**Figure 1:**
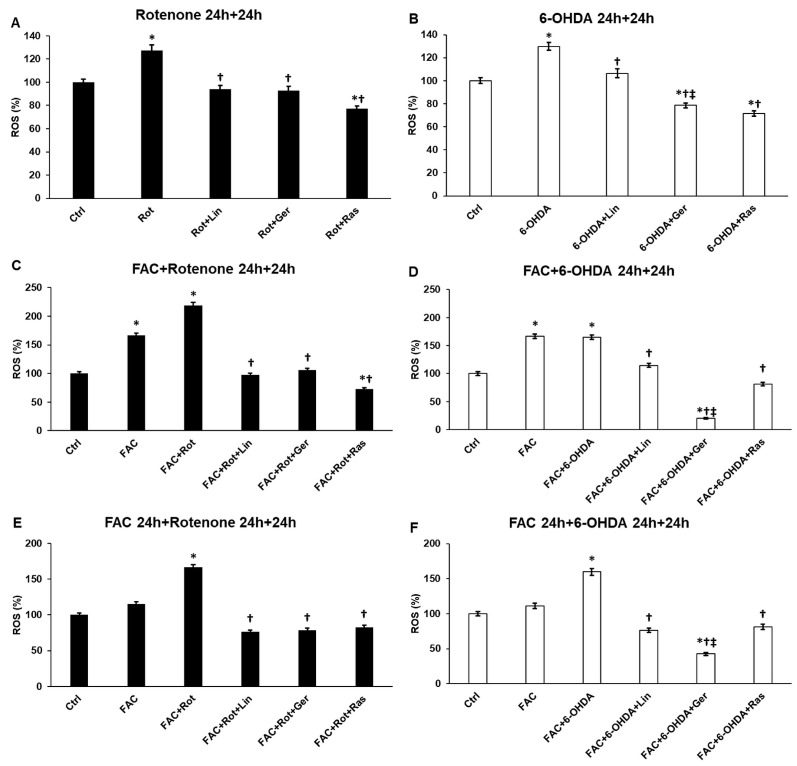
Reactive oxygen species production of the rotenone and 6-OHDA-induced differentiated SH-SY5Y cells. ROS was measured using a Fluorometric Intracellular ROS kit and was expressed as a percentage of the control cells’ production. (**A**) The cells were treated with rotenone for 24 h, followed by DMSO (in the case of Ctrl and Rot), linalool (Rot + Lin), geraniol (Rot + Ger), and rasagiline (Rot + Ras) for 24 h. (**B**) The cells were treated with 6-OHDA for 24 h, followed by DMSO (in the case of Ctrl and 6-OHDA), linalool (6-OHDA + Lin), geraniol (6-OHDA + Ger), and rasagiline (6-OHDA +Ras) for 24 h. (**C**) The cells were treated with ferric ammonium citrate (FAC) together with rotenone for 24 h, which was followed by DMSO (in the case of Ctrl, FAC, and FAC + Rot), linalool (FAC + Rot + Lin), geraniol (FAC + Rot + Ger), and rasagiline (FAC + Rot + Ras) for 24 h. (**D**) The cells were treated with ferric ammonium citrate (FAC) together with 6-OHDA for 24 h, which was followed by DMSO (in the case of Ctrl, FAC, and FAC + 6-OHDA), linalool (FAC + 6-OHDA + Lin), geraniol (FAC + 6-OHDA + Ger), and rasagiline (FAC + 6-OHDA + Ras) for 24 h. (**E**) The cells were pretreated with ferric ammonium citrate (FAC) for 24 h and then were treated with rotenone for additional 24 h, which was followed by DMSO (in the case of Ctrl, FAC, and FAC + Rot), linalool (FAC + Rot + Lin), geraniol (FAC + Rot + Ger), and rasagiline (FAC + Rot + Ras) for 24 h. (**F**) The cells were pretreated with ferric ammonium citrate (FAC) for 24 h and then were treated with 6-OHDA for additional 24 h, which was followed by DMSO (in the case of Ctrl, FAC, and FAC + 6-OHDA), linalool (FAC + 6-OHDA + Lin), geraniol (FAC + 6-OHDA + Ger), and rasagiline (FAC + 6-OHDA + Ras) for 24 h. The columns represent the mean value ± SD of three independent experiments. The number of technical replicates was four in each experiment. The asterisk shows *p* < 0.05 compared to the control. The cross indicates *p* < 0.05 compared to the rotenone or 6-OHDA treatments. The double cross signs indicate *p* < 0.05 compared to linalool and/or geraniol treatments.

The production of reactive oxygen species (ROS) reflects the rate of oxidative stress in the cells. In the case of neurodegenerative diseases, oxidative stress is one of the symptoms of the disease, leading to decreased viability. In our experiments, rotenone and 6-OHDA significantly increased intracellular ROS production (Figure 1A,B). In the presence of iron (FAC), elevated ROS production was observed, which was further improved by rotenone (Figure 1C) and by both rotenone and 6-OHDA in the case of FAC pretreatment (Figure 1E,F). Considering the effects of linalool and geraniol, it is evident that both essential oil components could reduce ROS production (Figure 1).

Comparing the two components, geraniol was more efficient in decreasing ROS when the cells were treated with 6-OHDA. However, the ROS levels were similar when rotenone or 6-OHDA was applied in the different experiments. Moreover, geraniol was significantly more potent than rasagiline in 6-OHDA treatments (Figure 1B,D,F). The different ROS values may be due to the different mechanisms of action of rotenone and 6-OHDA.

### 3.2. Linalool and Geraniol Influence the Small-Molecule Antioxidant Capacity of the Differentiated SH-SY5Y Cells

**Figure 2:**
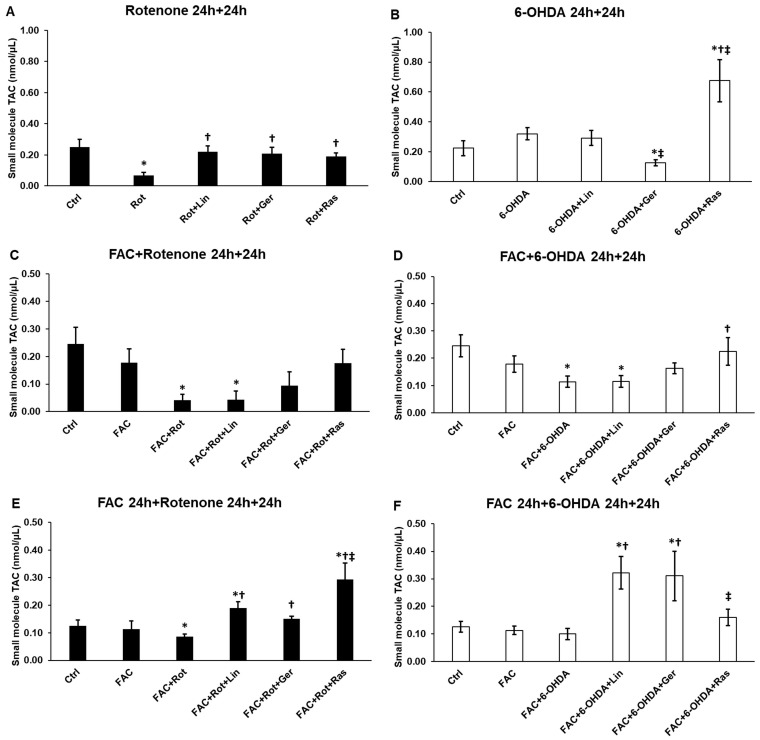
Small-molecule antioxidant capacity of the treated cells. The antioxidant capacity was measured using a Total Antioxidant Capacity Assay kit and Protein Mask and was expressed as nmol/μL. (**A**) The cells were treated with rotenone for 24 h, followed by DMSO (in the case of Ctrl and Rot), linalool (Rot + Lin), geraniol (Rot + Ger), and rasagiline (Rot + Ras) for 24 h. (**B**) The cells were treated with 6-OHDA for 24 h, followed by DMSO (in the case of Ctrl and 6-OHDA), linalool (6-OHDA + Lin), geraniol (6-OHDA + Ger), and rasagiline (6-OHDA +Ras) for 24 h. (**C**) The cells were treated with ferric ammonium citrate (FAC) together with rotenone for 24 h, which was followed by DMSO (in the case of Ctrl, FAC, and FAC + Rot), linalool (FAC + Rot + Lin), geraniol (FAC + Rot + Ger), and rasagiline (FAC + Rot + Ras) for 24 h. (**D**) The cells were treated with ferric ammonium citrate (FAC) together with 6-OHDA for 24 h, which was followed by DMSO (in the case of Ctrl, FAC, and FAC + 6-OHDA), linalool (FAC + 6-OHDA + Lin), geraniol (FAC + 6-OHDA + Ger), and rasagiline (FAC + 6-OHDA + Ras) for 24 h. (**E**) The cells were pretreated with ferric ammonium citrate (FAC) for 24 h and then were treated with rotenone for an additional 24 h, which was followed by DMSO (in the case of Ctrl, FAC, and FAC + Rot), linalool (FAC + Rot + Lin), geraniol (FAC + Rot + Ger), and rasagiline (FAC + Rot + Ras) for 24 h. (**F**) The cells were pretreated with ferric ammonium citrate (FAC) for 24 h and then were treated with 6-OHDA for additional 24 h, which was followed by DMSO (in the case of Ctrl, FAC, and FAC + 6-OHDA), linalool (FAC + 6-OHDA + Lin), geraniol (FAC + 6-OHDA + Ger), and rasagiline (FAC + 6-OHDA + Ras) for 24 h. The columns represent the mean value ± SD of three independent experiments. There were three technical replicates in each experiment. The asterisk shows *p* < 0.05 compared to the control. The cross indicates *p* < 0.05 compared to the rotenone or 6-OHDA treatments. The double cross signs indicate *p* < 0.05 compared to linalool and/or geraniol treatments.

Since linalool and geraniol are protective molecules against ROS production, their effects were measured on the antioxidant capacities of the cells. The small-molecule antioxidant capacity (SMAC) decreased when only rotenone was added to the cells. Adding linalool, geraniol, or rasagiline resulted in a significant elevation of SMAC (Figure 2A). When the cells were co-treated with rotenone and FAC, SMAC was also reduced. Still, only linalool and rasagiline were able to restore it. However, they could not increase SMAC to the control level (Figure 2C). In the case of FAC pretreatment, all three treatments elevated SMAC compared to the rotenone treatment (Figure 2E).

The 6-OHDA treatment alone did not reduce the SMAC level. Moreover, geraniol significantly decreased SMAC, while rasagiline significantly increased it. When FAC was added with 6-OHDA to the SH-SY5Y cells, the SMAC level was depleted. Only rasagiline could restore it near the control level (Figure 2D). Interestingly, in the case of FAC pretreatment, both linalool and geraniol significantly elevated SMAC compared to the 6-OHDA treatment (Figure 2F), suggesting that intracellular iron availability is implicated in the action of the essential oil compounds on the SMAC levels.

### 3.3. Linalool and Geraniol Have an Impact on the Protein Antioxidant Capacity of the Differentiated SH-SY5Y Cells

**Figure 3:**
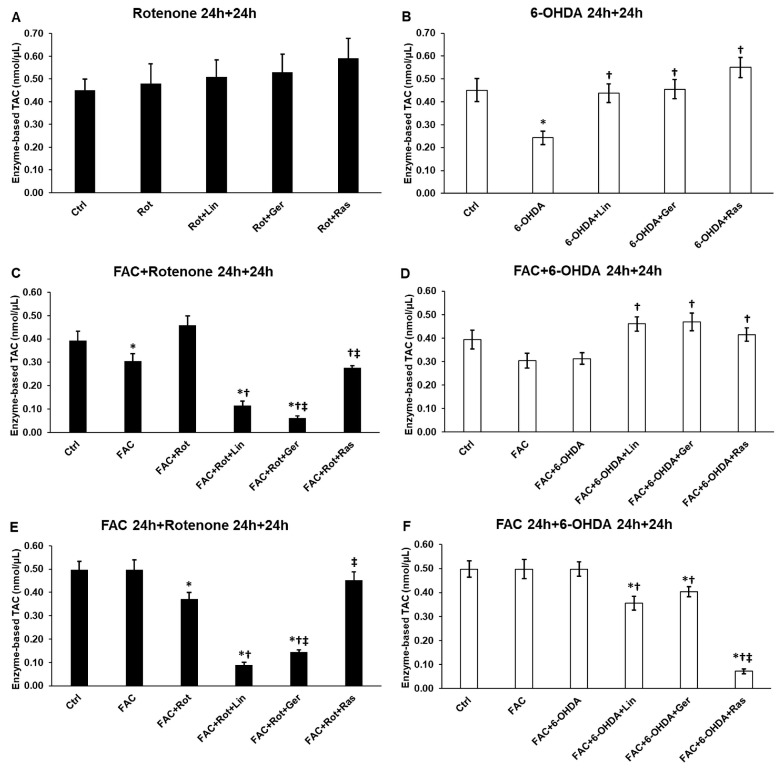
Concentration determination of the protein antioxidants in the treated cells. The protein antioxidant capacity was calculated from the total antioxidant capacity minus the small-molecule antioxidant capacity using the Total Antioxidant Capacity Assay kit and was expressed as nmol/μL. (**A**) The cells were treated with rotenone for 24 h, followed by DMSO (in the case of Ctrl and Rot), linalool (Rot + Lin), geraniol (Rot + Ger), and rasagiline (Rot + Ras) for 24 h. (**B**) The cells were treated with 6-OHDA for 24 h, followed by DMSO (in the case of Ctrl and 6-OHDA), linalool (6-OHDA + Lin), geraniol (6-OHDA + Ger), and rasagiline (6-OHDA + Ras) for 24 h. (**C**) The cells were treated with ferric ammonium citrate (FAC) together with rotenone for 24 h, which was followed by DMSO (in the case of Ctrl, FAC, and FAC + Rot), linalool (FAC + Rot + Lin), geraniol (FAC + Rot + Ger), and rasagiline (FAC + Rot + Ras) for 24 h. (**D**) The cells were treated with ferric ammonium citrate (FAC) together with 6-OHDA for 24 h, which was followed by DMSO (in the case of Ctrl, FAC, and FAC + 6-OHDA), linalool (FAC + 6-OHDA + Lin), geraniol (FAC + 6-OHDA + Ger), and rasagiline (FAC + 6-OHDA + Ras) for 24 h. (**E**) The cells were pretreated with ferric ammonium citrate (FAC) for 24 h and then were treated with rotenone for additional 24 h, which was followed by DMSO (in the case of Ctrl, FAC, and FAC + Rot), linalool (FAC + Rot + Lin), geraniol (FAC + Rot + Ger), and rasagiline (FAC + Rot + Ras) for 24 h. (**F**) The cells were pretreated with ferric ammonium citrate (FAC) for 24 h and then were treated with 6-OHDA for additional 24 h, which was followed by DMSO (in the case of Ctrl, FAC, and FAC + 6-OHDA), linalool (FAC + 6-OHDA + Lin), geraniol (FAC + 6-OHDA + Ger), and rasagiline (FAC + 6-OHDA + Ras) for 24 h. The columns represent the mean value ± SD of three independent experiments. The number of technical replicates was three in each experiment. The asterisk shows *p* < 0.05 compared to the control. The cross indicates *p* < 0.05 compared to the rotenone or 6-OHDA treatments. The double cross signs indicate *p* < 0.05 compared to linalool and/or geraniol treatments.

The protein antioxidant capacity (PAC) of the differentiated SH-SY5Y cells showed elevated levels in the case of rotenone treatment and after adding the essential oil compounds and rasagiline (Figure 3A), although these alterations were insignificant. Using 6-OHDA treatment, PAC significantly decreased, while linalool, geraniol, and rasagiline increased it (Figure 3B). When FAC was added with rotenone, PAC was slightly raised. After the treatment with linalool or geraniol, PAC levels were significantly reduced, which was enhanced in the case of geraniol treatment (Figure 3C). Interestingly, in the case of FAC and 6-OHDA co-treatments, opposite results were revealed; both essential oil components significantly increased PAC compared to the FAC + 6-OHDA-treated cells (Figure 3D). When the cells were pretreated with FAC, rotenone lowered PAC, and similarly to the co-treatments, both compounds significantly decreased PAC levels compared to rotenone, still in the opposite direction (Figure 3E).

We also revealed the opposite action of the essential oil components when FAC pretreatment was used before the addition of 6-OHDA, compared to the FAC + 6-OHDA co-treatments (Figure 3F). In the case of FAC pretreatment, linalool and geraniol significantly abated the PAC levels as well as rasagiline (Figure 3F). Based on the results, it is supposed that the iron availability both intra- and extracellularly affects the PAC levels.

### 3.4. Linalool Decreases the IL-6 Secretion of the Differentiated SH-SY5Y Cells

**Figure 4:**
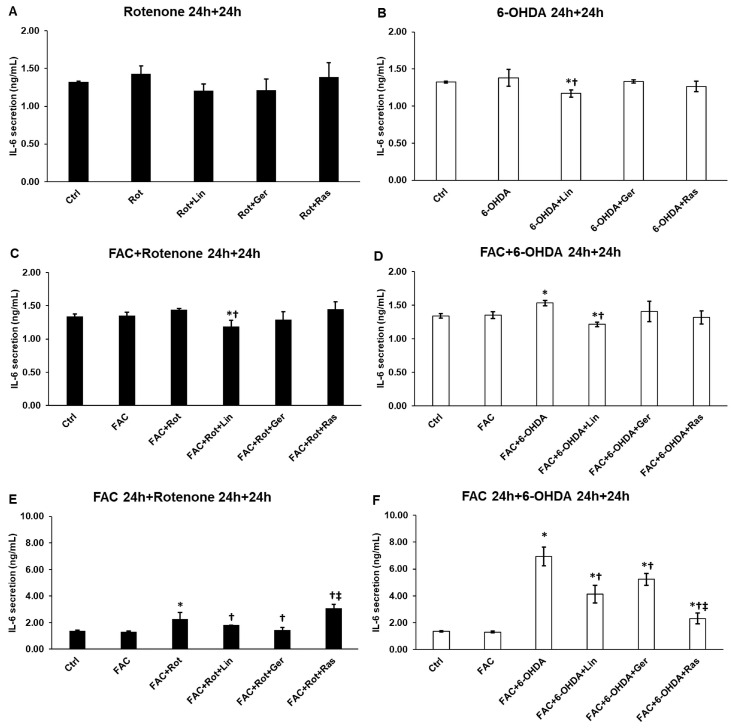
Secretion measurements of IL-6 pro-inflammatory cytokine from the treated SH-SY5Y cells. The IL-6 cytokine concentration was determined from the cell culture supernatants using a human IL-6 ELISA Kit. (**A**) The cells were treated with rotenone for 24 h, followed by DMSO (in the case of Ctrl and Rot), linalool (Rot + Lin), geraniol (Rot + Ger), and rasagiline (Rot + Ras) for 24 h. (**B**) The cells were treated with 6-OHDA for 24 h, followed by DMSO (in the case of Ctrl and 6-OHDA), linalool (6-OHDA + Lin), geraniol (6-OHDA + Ger), and rasagiline (6-OHDA +Ras) for 24 h. (**C**) The cells were treated with ferric ammonium citrate (FAC) together with rotenone for 24 h, which was followed by DMSO (in the case of Ctrl, FAC, and FAC + Rot), linalool (FAC + Rot + Lin), geraniol (FAC + Rot + Ger), and rasagiline (FAC + Rot + Ras) for 24 h. (**D**) The cells were treated with ferric ammonium citrate (FAC) together with 6-OHDA for 24 h, which was followed by DMSO (in the case of Ctrl, FAC, and FAC + 6-OHDA), linalool (FAC + 6-OHDA + Lin), geraniol (FAC + 6-OHDA + Ger), and rasagiline (FAC + 6-OHDA + Ras) for 24 h. (**E**) The cells were pretreated with ferric ammonium citrate (FAC) for 24 h and then were treated with rotenone for additional 24 h, which was followed by DMSO (in the case of Ctrl, FAC, and FAC + Rot), linalool (FAC + Rot + Lin), geraniol (FAC + Rot + Ger), and rasagiline (FAC + Rot + Ras) for 24 h. (**F**) The cells were pretreated with ferric ammonium citrate (FAC) for 24 h and then were treated with 6-OHDA for an additional 24 h, which was followed by DMSO (in the case of Ctrl, FAC, and FAC + 6-OHDA), linalool (FAC + 6-OHDA + Lin), geraniol (FAC + 6-OHDA + Ger), and rasagiline (FAC + 6-OHDA + Ras) for 24 h. The columns represent the mean value ± SD of three independent experiments. The number of technical replicates was three in each experiment. The asterisk shows *p* < 0.05 compared to the control. The cross indicates *p* < 0.05 compared to the rotenone or 6-OHDA treatments. The double cross signs indicate *p* < 0.05 compared to linalool and/or geraniol treatments.

Next, the anti-inflammatory effects of linalool and geraniol were determined in the in vitro Parkinson’s model. Both rotenone and 6-OHDA increased the IL-6 secretions, but significant alterations were only found when FAC was used together with 6-OHDA or the cells were pretreated with FAC (Figure 4C,E,F). Linalool treatment reduced the IL-6 production of the differentiated SH-SY5Y cells, but it was more efficient in the case of 6-OHDA treatments (Figure 4B,D,F). Geraniol was less effective than linalool but still significantly decreased IL-6 secretion in the case of FAC-pretreated cells (Figure 4E,F).

### 3.5. Effects of Essential Oil Compounds on the IL-1β Secretion

**Figure 5:**
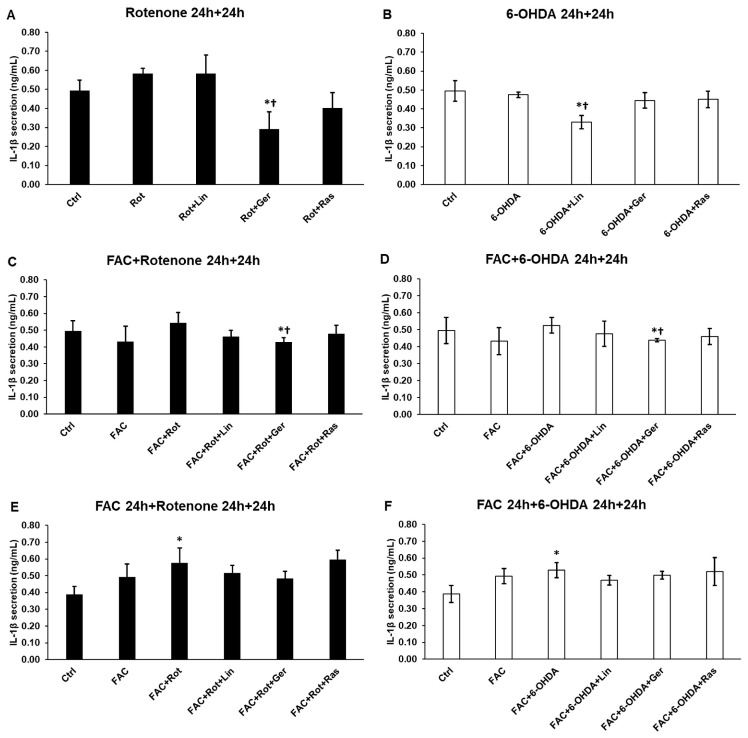
Secretion measurements of IL-1β pro-inflammatory cytokine from the treated SH-SY5Y cells. The IL-1β cytokine concentration was determined from the cell culture supernatants using a human IL-6 ELISA Kit. (**A**) The cells were treated with rotenone for 24 h, followed by DMSO (in the case of Ctrl and Rot), linalool (Rot + Lin), geraniol (Rot + Ger), and rasagiline (Rot + Ras) for 24 h. (**B**) The cells were treated with 6-OHDA for 24 h, followed by DMSO (in the case of Ctrl and 6-OHDA), linalool (6-OHDA + Lin), geraniol (6-OHDA + Ger), and rasagiline (6-OHDA +Ras) for 24 h. (**C**) The cells were treated with ferric ammonium citrate (FAC) together with rotenone for 24 h, which was followed by DMSO (in the case of Ctrl, FAC, and FAC + Rot), linalool (FAC + Rot + Lin), geraniol (FAC + Rot + Ger), and rasagiline (FAC + Rot + Ras) for 24 h. (**D**) The cells were treated with ferric ammonium citrate (FAC) together with 6-OHDA for 24 h, which was followed by DMSO (in the case of Ctrl, FAC, and FAC + 6-OHDA), linalool (FAC + 6-OHDA + Lin), geraniol (FAC + 6-OHDA + Ger), and rasagiline (FAC + 6-OHDA + Ras) for 24 h. (**E**) The cells were pretreated with ferric ammonium citrate (FAC) for 24 h and then were treated with rotenone for additional 24 h, which was followed by DMSO (in the case of Ctrl, FAC, and FAC + Rot), linalool (FAC + Rot + Lin), geraniol (FAC + Rot + Ger), and rasagiline (FAC + Rot + Ras) for 24 h. (**F**) The cells were pretreated with ferric ammonium citrate (FAC) for 24 h and then were treated with 6-OHDA for an additional 24 h, which was followed by DMSO (in the case of Ctrl, FAC, and FAC + 6-OHDA), linalool (FAC + 6-OHDA + Lin), geraniol (FAC + 6-OHDA + Ger), and rasagiline (FAC + 6-OHDA + Ras) for 24 h. The columns represent the mean value ± SD of three independent experiments. The number of technical replicates was three in each experiment. The asterisk shows *p* < 0.05 compared to the control. The cross indicates *p* < 0.05 compared to the rotenone or 6-OHDA treatments.

In the case of IL-1β, a significant elevation was found only when the cells were pretreated with FAC before rotenone or 6-OHDA (Figure 5E,F). Interestingly, in the case of rotenone treatment, only geraniol was, but in the case of 6-OHDA treatment, only linalool was able to reduce the IL-1β production (Figure 5A,B). When rotenone or 6-OHDA were added to the cells with FAC, only geraniol decreased the IL-1β levels (Figure 5C,D). In the case of FAC pretreatment, a minor reduction was observed in the case of both essential oil compounds (Figure 5E,F).

### 3.6. Linalool and Geraniol Reduced the Secretion of IL-8 in the Differentiated SH-SY5Y Cells

**Figure 6:**
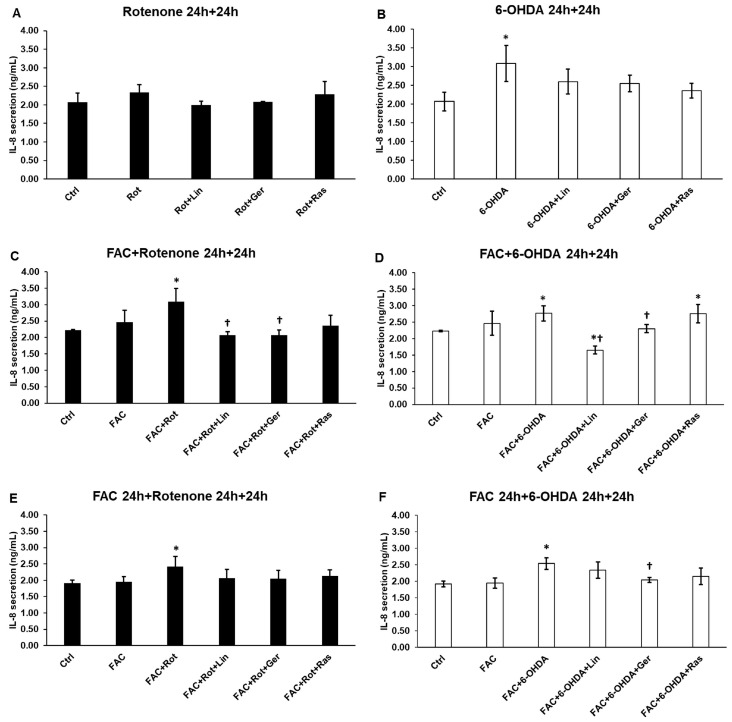
Secretion measurements of IL-8 from the treated SH-SY5Y cells. The IL-8 cytokine concentration was determined from the cell culture supernatants using a human IL-6 ELISA Kit. (**A**) The cells were treated with rotenone for 24 h, followed by DMSO (in the case of Ctrl and Rot), linalool (Rot + Lin), geraniol (Rot + Ger), and rasagiline (Rot + Ras) for 24 h. (**B**) The cells were treated with 6-OHDA for 24 h, followed by DMSO (in the case of Ctrl and 6-OHDA), linalool (6-OHDA + Lin), geraniol (6-OHDA + Ger), and rasagiline (6-OHDA +Ras) for 24 h. (**C**) The cells were treated with ferric ammonium citrate (FAC) together with rotenone for 24 h, which was followed by DMSO (in the case of Ctrl, FAC, and FAC + Rot), linalool (FAC + Rot + Lin), geraniol (FAC + Rot + Ger), and rasagiline (FAC + Rot + Ras) for 24 h. (**D**) The cells were treated with ferric ammonium citrate (FAC) together with 6-OHDA for 24 h, which was followed by DMSO (in the case of Ctrl, FAC, and FAC + 6-OHDA), linalool (FAC + 6-OHDA + Lin), geraniol (FAC + 6-OHDA + Ger), and rasagiline (FAC + 6-OHDA + Ras) for 24 h. (**E**) The cells were pretreated with ferric ammonium citrate (FAC) for 24 h and then were treated with rotenone for additional 24 h, which was followed by DMSO (in the case of Ctrl, FAC, and FAC + Rot), linalool (FAC + Rot + Lin), geraniol (FAC + Rot + Ger), and rasagiline (FAC + Rot + Ras) for 24 h. (**F**) The cells were pretreated with ferric ammonium citrate (FAC) for 24 h and then were treated with 6-OHDA for an additional 24 h, which was followed by DMSO (in the case of Ctrl, FAC, and FAC + 6-OHDA), linalool (FAC + 6-OHDA + Lin), geraniol (FAC + 6-OHDA + Ger), and rasagiline (FAC + 6-OHDA + Ras) for 24 h. The columns represent the mean value ± SD of three independent experiments. The number of technical replicates was three in each experiment. The asterisk shows *p* < 0.05 compared to the control. The cross indicates *p* < 0.05 compared to the rotenone or 6-OHDA treatments.

Both essential oil components caused a marginal decrease in the IL-8 secretion after rotenone and 6-OHDA treatments (Figure 6A,B). When iron was present in the culture medium, linalool and geraniol significantly reduced the IL-8 production of the differentiated SH-SY5Y cells (Figure 6C,D). Again, in the case of FAC pretreatment, only slight alterations were found after adding the essential oil components (Figure 6E,F).

### 3.7. Effects of Essential Oil Compounds on the Fractalkine Secretion

**Figure 7:**
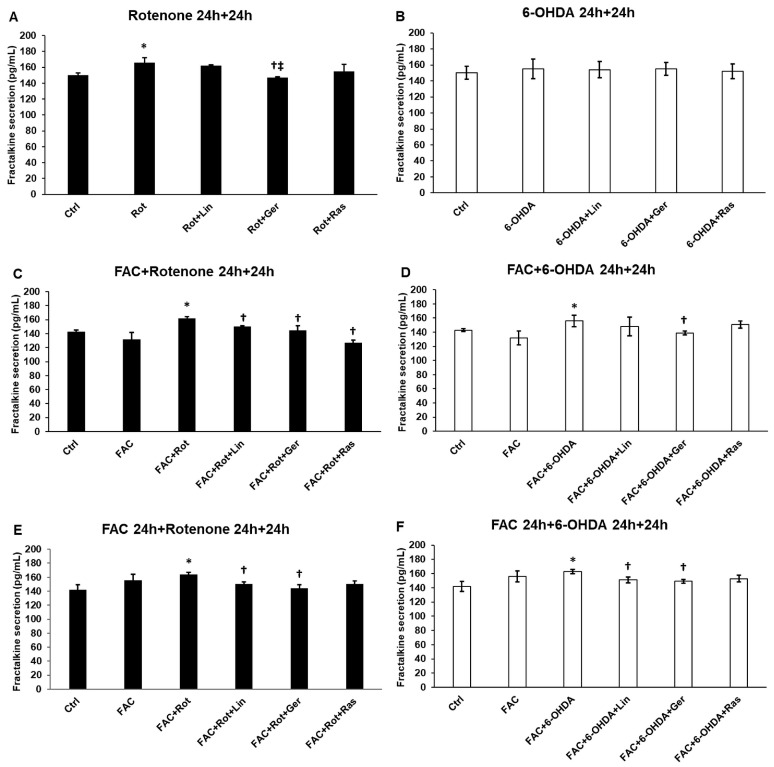
Secretion measurements of fractalkine from the treated SH-SY5Y cells. The FKN concentration was determined from the cell culture supernatants using a human IL-6 ELISA Kit. (**A**) The cells were treated with rotenone for 24 h, followed by DMSO (in the case of Ctrl and Rot), linalool (Rot + Lin), geraniol (Rot + Ger), and rasagiline (Rot + Ras) for 24 h. (**B**) The cells were treated with 6-OHDA for 24 h, followed by DMSO (in the case of Ctrl and 6-OHDA), linalool (6-OHDA + Lin), geraniol (6-OHDA + Ger), and rasagiline (6-OHDA +Ras) for 24 h. (**C**) The cells were treated with ferric ammonium citrate (FAC) together with rotenone for 24 h, which was followed by DMSO (in the case of Ctrl, FAC, and FAC + Rot), linalool (FAC + Rot + Lin), geraniol (FAC + Rot + Ger), and rasagiline (FAC + Rot + Ras) for 24 h. (**D**) The cells were treated with ferric ammonium citrate (FAC) together with 6-OHDA for 24 h, which was followed by DMSO (in the case of Ctrl, FAC, and FAC + 6-OHDA), linalool (FAC + 6-OHDA + Lin), geraniol (FAC + 6-OHDA + Ger), and rasagiline (FAC + 6-OHDA + Ras) for 24 h. (**E**) The cells were pretreated with ferric ammonium citrate (FAC) for 24 h and then were treated with rotenone for additional 24 h, which was followed by DMSO (in the case of Ctrl, FAC, and FAC + Rot), linalool (FAC + Rot + Lin), geraniol (FAC + Rot + Ger), and rasagiline (FAC + Rot + Ras) for 24 h. (**F**) The cells were pretreated with ferric ammonium citrate (FAC) for 24 h and then were treated with 6-OHDA for an additional 24 h, which was followed by DMSO (in the case of Ctrl, FAC, and FAC + 6-OHDA), linalool (FAC + 6-OHDA + Lin), geraniol (FAC + 6-OHDA + Ger), and rasagiline (FAC + 6-OHDA + Ras) for 24 h. The columns represent the mean value ± SD of three independent experiments. The number of technical replicates was three in each experiment. The asterisk shows *p* < 0.05 compared to the control. The cross indicates *p* < 0.05 compared to the rotenone or 6-OHDA treatments. The double cross signs indicate *p* < 0.05 compared to linalool and/or geraniol treatments.

Fractalkine secretion is a valuable marker of neuronal damage or neuroinflammation. It was found that fractalkine secretion was only triggered by rotenone and 6-OHDA when iron was present (Figure 7C–F). Furthermore, the attenuating effect of linalool and geraniol was also revealed when FAC was added to the cells, suggesting a potential role of iron in regulating fractalkine or in the mechanism of action of the essential oil compounds (Figure 7C–F).

### 3.8. The Effects of Linalool and Geraniol on the ATP Levels of Differentiated SH-SY5Y Cells

**Figure 8:**
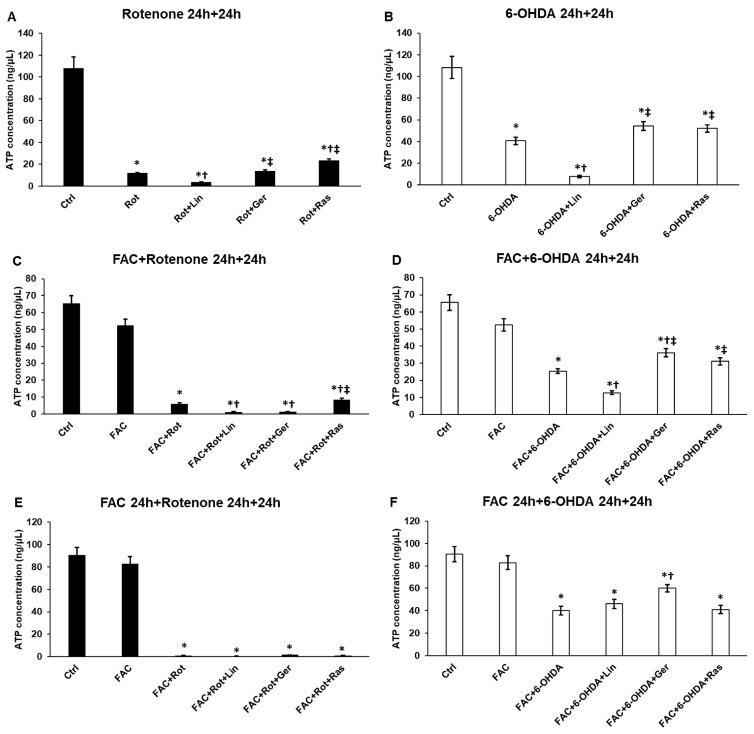
ATP concentration measurements from the treated cells. The ATP concentration was determined using an ATP assay kit according to the manufacturer’s protocol. The values were expressed as ng/μL. (**A**) The cells were treated with rotenone for 24 h, followed by DMSO (in the case of Ctrl and Rot), linalool (Rot + Lin), geraniol (Rot + Ger), and rasagiline (Rot + Ras) for 24 h. (**B**) The cells were treated with 6-OHDA for 24 h, followed by DMSO (in the case of Ctrl and 6-OHDA), linalool (6-OHDA + Lin), geraniol (6-OHDA + Ger), and rasagiline (6-OHDA +Ras) for 24 h. (**C**) The cells were treated with ferric ammonium citrate (FAC) together with rotenone for 24 h, which was followed by DMSO (in the case of Ctrl, FAC, and FAC + Rot), linalool (FAC + Rot + Lin), geraniol (FAC + Rot + Ger), and rasagiline (FAC + Rot + Ras) for 24 h. (**D**) The cells were treated with ferric ammonium citrate (FAC) together with 6-OHDA for 24 h, which was followed by DMSO (in the case of Ctrl, FAC, and FAC + 6-OHDA), linalool (FAC + 6-OHDA + Lin), geraniol (FAC + 6-OHDA + Ger), and rasagiline (FAC + 6-OHDA + Ras) for 24 h. (**E**) The cells were pretreated with ferric ammonium citrate (FAC) for 24 h and then were treated with rotenone for additional 24 h, which was followed by DMSO (in the case of Ctrl, FAC, and FAC + Rot), linalool (FAC + Rot + Lin), geraniol (FAC + Rot + Ger), and rasagiline (FAC + Rot + Ras) for 24 h. (**F**) The cells were pretreated with ferric ammonium citrate (FAC) for 24 h and then were treated with 6-OHDA for an additional 24 h, which was followed by DMSO (in the case of Ctrl, FAC, and FAC + 6-OHDA), linalool (FAC + 6-OHDA + Lin), geraniol (FAC + 6-OHDA + Ger), and rasagiline (FAC + 6-OHDA + Ras) for 24 h. The columns represent the mean value ± SD of three independent experiments. The number of technical replicates was three in each experiment. The asterisk shows *p* < 0.05 compared to the control. The cross indicates *p* < 0.05 compared to the rotenone or 6-OHDA treatments. The double cross signs indicate *p* < 0.05 compared to linalool and/or geraniol treatments.

Our research has uncovered significant findings on the effects of rotenone and 6-OHDA treatments on ATP levels in differentiated SH-SY5Y cells (Figure 8). The rotenone treatment, known for inhibiting complex I in the mitochondrial membrane, significantly reduced ATP production. Interestingly, neither linalool nor geraniol could counteract this effect. In the case of 6-OHDA treatments, a decrease in ATP levels was also observed. However, geraniol emerged as a potential solution, effectively improving intracellular ATP concentrations. On the other hand, linalool seemed to have a more detrimental effect on ATP production, hinting at a different working mechanism for linalool.

### 3.9. Distinct Effects of Essential Oil Compounds on the Intracellular Iron Content and Ferritin Heavy Chain Expression

**Figure 9:**
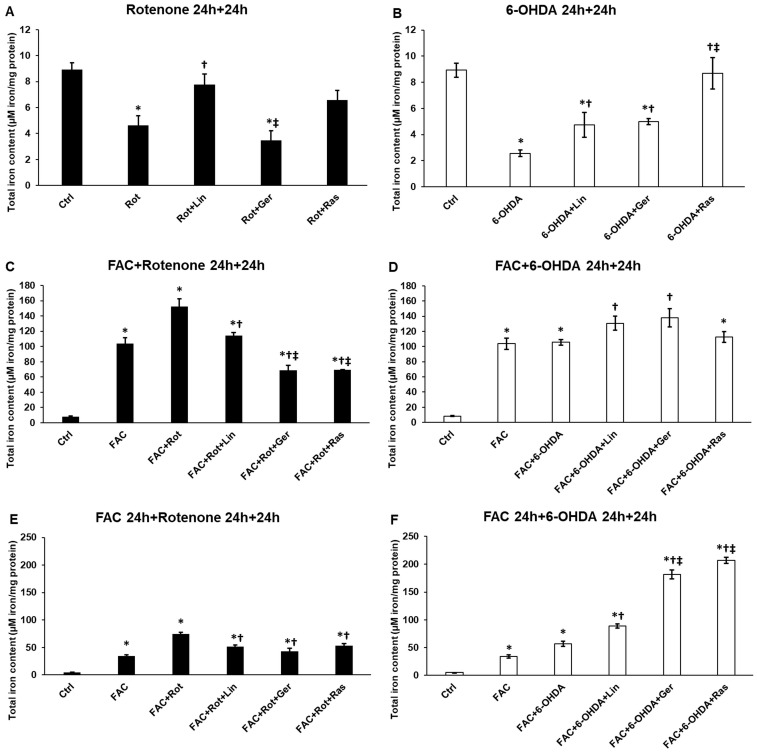
The total iron content of the variously treated differentiated SH-SY5Y cells. The total iron content was determined from the whole-cell lysate by a ferrozine-based colorimetric method. For normalization, the protein concentration of the samples was used. The iron content was expressed as μM iron/mg protein. (**A**) The cells were treated with rotenone for 24 h, followed by DMSO (in the case of Ctrl and Rot), linalool (Rot + Lin), geraniol (Rot + Ger), and rasagiline (Rot + Ras) for 24 h. (**B**) The cells were treated with 6-OHDA for 24 h, followed by DMSO (in the case of Ctrl and 6-OHDA), linalool (6-OHDA + Lin), geraniol (6-OHDA + Ger), and rasagiline (6-OHDA +Ras) for 24 h. (**C**) The cells were treated with ferric ammonium citrate (FAC) together with rotenone for 24 h, which was followed by DMSO (in the case of Ctrl, FAC, and FAC + Rot), linalool (FAC + Rot + Lin), geraniol (FAC + Rot + Ger), and rasagiline (FAC + Rot + Ras) for 24 h. (**D**) The cells were treated with ferric ammonium citrate (FAC) together with 6-OHDA for 24 h, which was followed by DMSO (in the case of Ctrl, FAC, and FAC + 6-OHDA), linalool (FAC + 6-OHDA + Lin), geraniol (FAC + 6-OHDA + Ger), and rasagiline (FAC + 6-OHDA + Ras) for 24 h. (**E**) The cells were pretreated with ferric ammonium citrate (FAC) for 24 h and then were treated with rotenone for an additional 24 h, which was followed by DMSO (in the case of Ctrl, FAC, and FAC + Rot), linalool (FAC + Rot + Lin), geraniol (FAC + Rot + Ger), and rasagiline (FAC + Rot + Ras) for 24 h. (**F**) The cells were pretreated with ferric ammonium citrate (FAC) for 24 h and then were treated with 6-OHDA for an additional 24 h, which was followed by DMSO (in the case of Ctrl, FAC, and FAC + 6-OHDA), linalool (FAC + 6-OHDA + Lin), geraniol (FAC + 6-OHDA + Ger), and rasagiline (FAC + 6-OHDA + Ras) for 24 h. The columns represent the mean value ± SD of three independent experiments. The number of technical replicates was three in each experiment. The asterisk shows *p* < 0.05 compared to the control. The cross indicates *p* < 0.05 compared to the rotenone or 6-OHDA treatments. The double cross signs indicate *p* < 0.05 compared to linalool and/or geraniol treatments.

The previous results raise the possibility that the effects of essential oil compounds on iron may be interconnected. Therefore, the total iron content of the differently treated SH-SY5Y cells was determined. When the cells were treated only with rotenone or 6-OHDA, a significant reduction was observed in the total iron content (Figure 9A,B). On the other hand, when iron supplementation was utilized in the experiments, both rotenone and 6-OHDA promoted iron accumulation (Figure 9C–F).

In the case of rotenone treatment alone, linalool significantly increased the iron content (Figure 9A). When rotenone was used with FAC, both essential oil components significantly reduced the cellular iron level. Moreover, geraniol was more potent than linalool (Figure 9C). In the case of FAC pretreatment, both essential oil components lowered the iron content with the same efficiency (Figure 9E).

When 6-OHDA treatment was applied alone, both essential oil compounds raised the total iron content of the cells (Figure 9B). In the presence of iron during the 6-OHDA treatment, linalool and geraniol elevated the iron level compared to the 6-OHDA treatment. However, these changes were not significant (Figure 9D). Using FAC treatment before the addition of 6-OHDA to the cells, both linalool and geraniol augmented the total iron levels (Figure 9F). Interestingly, geraniol treatment led to twice as high iron levels as linalool treatment (Figure 9F).

**Figure 10:**
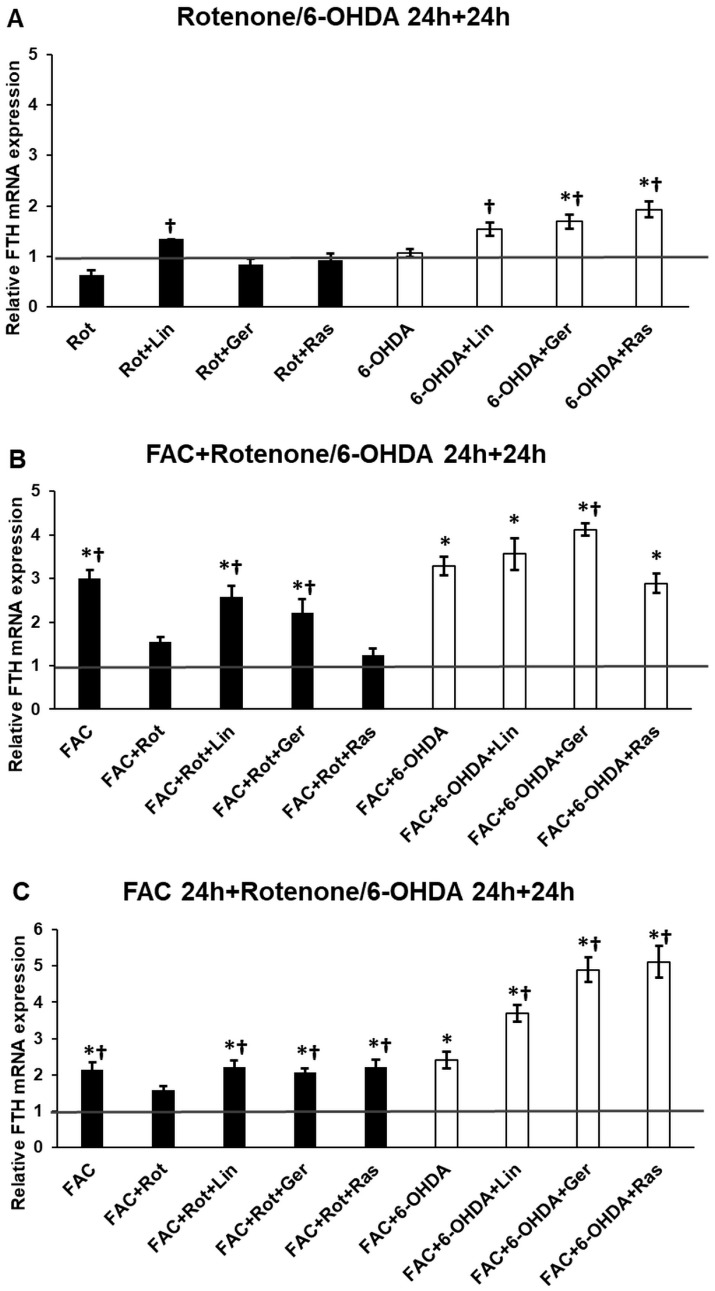
Real-time PCR analysis of the FTH gene of the treated cells. The real-time PCR was performed using a SYBR Green protocol. GAPDH was used as a normalization gene. The relative mRNA expression of the control cells was considered 1 (grey horizontal line). (**A**) The cells were treated with rotenone or 6-OHDA for 24 h, followed by DMSO (in the case of Ctrl and Rot), linalool (Rot + Lin), geraniol (Rot + Ger), and rasagiline (Rot + Ras) for 24 h. (**B**) The cells were treated with ferric ammonium citrate (FAC) together with rotenone or 6-OHDA for 24 h, which was followed by DMSO (in the case of Ctrl, FAC, and FAC + Rot), linalool (FAC + Rot + Lin), geraniol (FAC + Rot + Ger), and rasagiline (FAC + Rot + Ras) for 24 h. (**C**) The cells were pretreated with ferric ammonium citrate (FAC) for 24 h and then were treated with rotenone or 6-OHDA for an additional 24 h, which was followed by DMSO (in the case of Ctrl, FAC, and FAC + Rot), linalool (FAC + Rot + Lin), geraniol (FAC + Rot + Ger), and rasagiline (FAC + Rot + Ras) for 24 h. The columns represent the mean value ± SD of three independent experiments. The number of technical replicates was three in each experiment. The asterisk shows *p* < 0.05 compared to the control. The cross indicates *p* < 0.05 compared to the rotenone or 6-OHDA treatments.

Ferritin heavy chain is an antioxidant protein with ferroxidase activity, with which it can safely store iron and prevent form iron-mediated oxidative stress. The gene expression of FTH was examined to reveal whether the essential oil compounds influence it. According to the results, it is evident that iron availability influences the expression of FTH (Figure 10A). Rotenone decreased the FTH mRNA level compared to the FAC treatment (Figure 10B). At the same time, linalool and geraniol did not cause a significant reduction (Figure 10B,C). In the case of 6-OHDA treatments, no significant elevation was observed in the FTH expression, while both essential oil components significantly raised the FTH mRNA levels compared to the FAC treatments (Figure 10B,C). These results propose the protective effects of the essential oil components against iron-mediated oxidative stress.

### 3.10. Alterations of Iron-Related Genes after Linalool and Geraniol Treatments

Based on the alterations observed in the intracellular iron content, the mRNA expression of the iron-related genes responsible for heme degradation and working as an antioxidant enzyme (heme-oxygenase-1, HO-1), iron uptake (transferrin receptor 1, TfR1), and release (ferroportin, FP) were examined.

**Figure 11:**
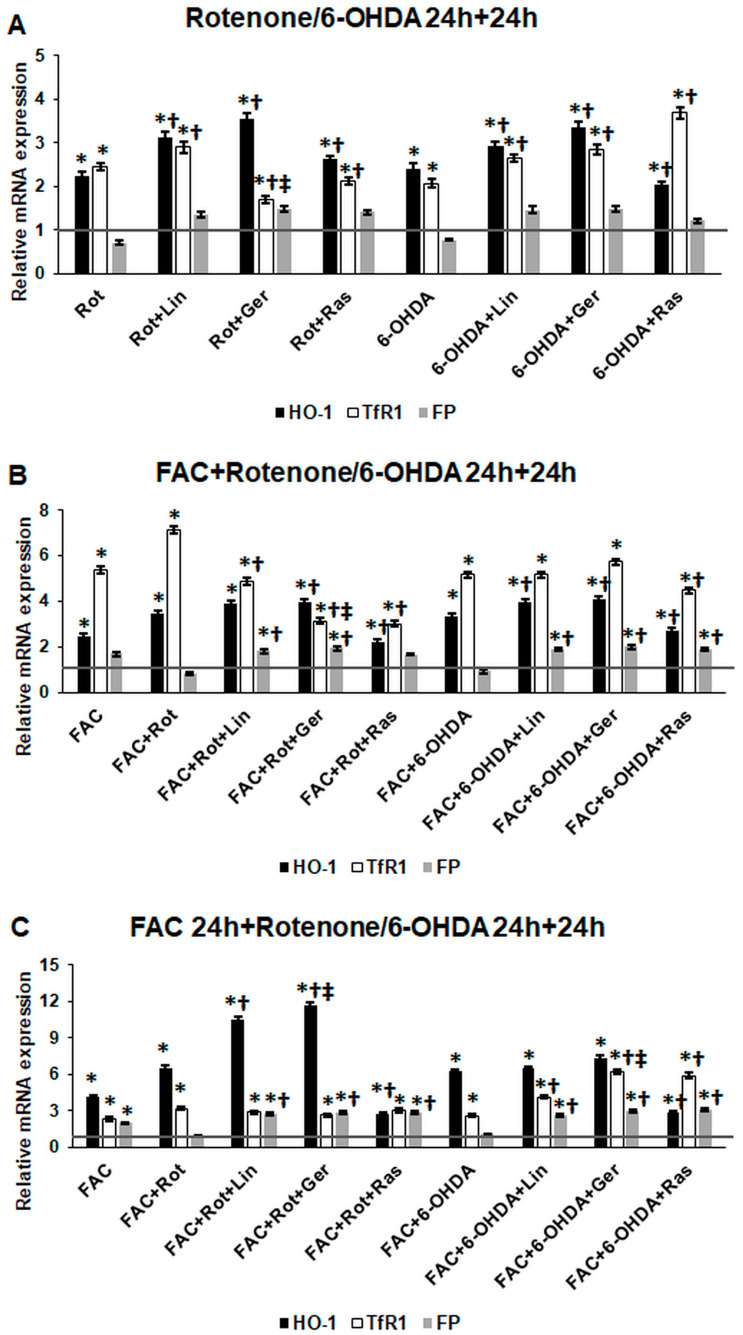
Real-time PCR analysis of the heme-oxygenase-1 (HO-1), transferrin receptor 1 (TfR1), and ferroportin (FP) genes of the treated cells. The real-time PCR was performed using a SYBR Green protocol. GAPDH was used as a housekeeping gene. The relative mRNA expression of the control cells was considered 1 (grey horizontal line). (**A**) The cells were treated with rotenone or 6-OHDA for 24 h, followed by DMSO (in the case of Ctrl and Rot), linalool (Rot + Lin), geraniol (Rot + Ger), and rasagiline (Rot + Ras) for 24 h. (**B**) The cells were treated with ferric ammonium citrate (FAC) together with rotenone or 6-OHDA for 24 h, which was followed by DMSO (in the case of Ctrl, FAC, and FAC + Rot), linalool (FAC + Rot + Lin), geraniol (FAC + Rot + Ger), and rasagiline (FAC + Rot + Ras) for 24 h (**C**) The cells were pretreated with ferric ammonium citrate (FAC) for 24 h and then were treated with rotenone or 6-OHDA for additional 24 h, which was followed by DMSO (in the case of Ctrl, FAC, and FAC + Rot), linalool (FAC + Rot + Lin), geraniol (FAC + Rot + Ger), and rasagiline (FAC + Rot + Ras) for 24 h. The columns represent the mean value ± SD of three independent experiments. The number of technical replicates was three in each experiment. The asterisk shows *p* < 0.05 compared to the control. The cross indicates *p* < 0.05 compared to the rotenone or 6-OHDA treatments. The double cross signs indicate *p* < 0.05 compared to linalool and/or geraniol treatments.

Both rotenone and 6-OHDA significantly increased HO-1 and TfR1 mRNA expressions compared to the controls (Figure 11A). Linalool significantly elevated the expression of HO-1 and TfR1 compared to rotenone or 6-OHDA treatment. In the case of geraniol addition, the HO-1 level was raised (Figure 11A). Meanwhile, an opposite action was revealed in TfR1 mRNA expression after rotenone treatment, with a reduction in the case of rotenone treatment but an increment after 6-OHDA treatment (Figure 11A). In the case of the iron exporter FP, rotenone and 6-OHDA caused a reduction. Still, the essential oil components triggered a moderate but insignificant elevation in the gene expression (Figure 11A).

When FAC was utilized with rotenone or 6-OHDA, both HO-1 and TfR1 showed higher mRNA expression levels than without FAC (Figure 11A,B). With the addition of linalool to the cells after rotenone treatment, the HO-1 level increased slightly. In contrast, the TfR1 mRNA level significantly decreased compared to that of the rotenone treatment (Figure 11B). Using geraniol after rotenone treatment, the HO-1 expression rate was raised considerably, but the TfR1 level was significantly reduced compared to the rotenone and linalool treatments (Figure 11B). The essential oil components provoked the augmentation of HO-1, and there were no alterations in the TfR1 mRNA expression levels after the 6-OHDA treatment (Figure 11B). Still, the essential oil components triggered a significant elevation in the gene expression of FP (Figure 11B).

After FAC pretreatment, The HO-1 and TfR1 mRNA expression significantly increased compared to the controls, and rotenone or 6-OHDA treatments elevated their levels more (Figure 11C). Linalool and geraniol raised the HO-1 expression levels. Still, they did not alter the TfR1 mRNA levels compared to the rotenone treatment (Figure 11C). In the case of FP, a significant elevation was observed. Using the essential oil after the 6-OHDA treatments, a significant magnification was revealed in TfR1 and FP mRNA levels but not in HO-1 levels (Figure 11C).

The assorted changes in the gene expression levels of HO-1, TfR1, and FP that occur in response to linalool and geraniol treatments may depend on the iron availability and the inducer (rotenone/6-OHDA) used in the experimental model.

### 3.11. The Essential Oil Compounds Modify the α-Synuclein Expression of the Differentiated SH-SY5Y Cells

**Figure 12:**
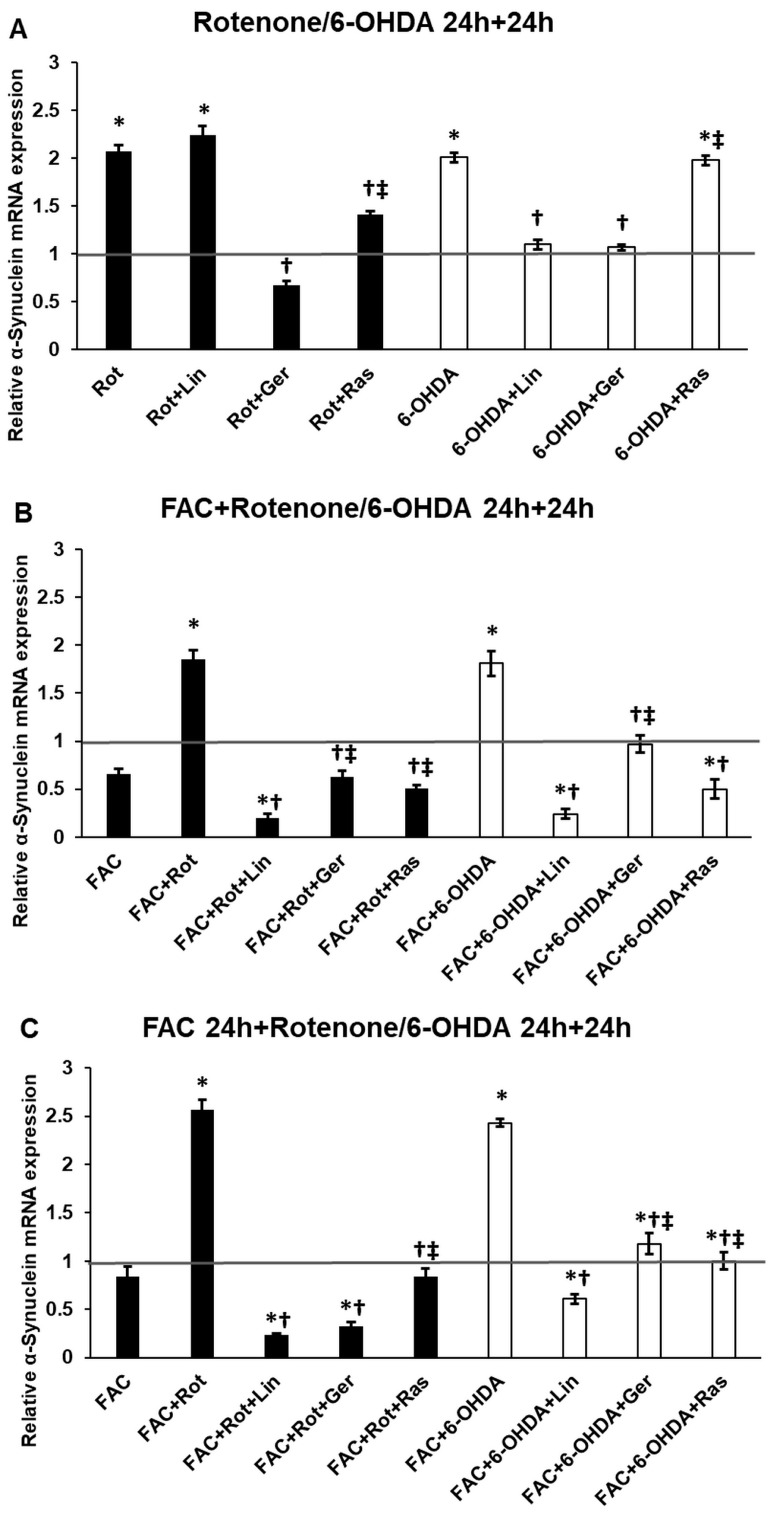
Real-time PCR analysis of the α-Synuclein gene of the treated cells. The real-time PCR was performed using a SYBR Green protocol. GAPDH was used as a normalization gene. The relative mRNA expression of the control cells was considered 1 (grey horizontal line). (**A**) The cells were treated with rotenone or 6-OHDA for 24 h, followed by DMSO (in the case of Ctrl and Rot), linalool (Rot + Lin), geraniol (Rot + Ger), and rasagiline (Rot + Ras) for 24 h. (**B**) The cells were treated with ferric ammonium citrate (FAC) together with rotenone or 6-OHDA for 24 h, which was followed by DMSO (in the case of Ctrl, FAC, and FAC + Rot), linalool (FAC + Rot + Lin), geraniol (FAC + Rot + Ger), and rasagiline (FAC + Rot + Ras) for 24 h (**C**) The cells were pretreated with ferric ammonium citrate (FAC) for 24 h and then were treated with rotenone or 6-OHDA for additional 24 h, which was followed by DMSO (in the case of Ctrl, FAC, and FAC + Rot), linalool (FAC + Rot + Lin), geraniol (FAC + Rot + Ger), and rasagiline (FAC + Rot + Ras) for 24 h. The columns represent the mean value ± SD of three independent experiments. The number of technical replicates was three in each experiment. The asterisk shows *p* < 0.05 compared to the control. The cross indicates *p* < 0.05 compared to the rotenone or 6-OHDA treatments. The double cross signs indicate *p* < 0.05 compared to linalool and/or geraniol treatments.

The α-synuclein mRNA expression levels were also examined in the in vitro Parkinson’s model to reveal the potential therapeutic impact of linalool, geraniol, or both. The rotenone and 6-OHDA treatments significantly increased the α-synuclein expression levels compared to the controls (Figure 12A–C). In the case of rotenone treatment, geraniol played a significant role in reducing the α-synuclein mRNA level (Figure 12A). Meanwhile, using 6-OHDA, both essential oil components demonstrated their potential to reduce the expression levels (Figure 12A).

In the presence of iron and rotenone, linalool was more efficient in lowering the α-synuclein expression compared to geraniol treatment (Figure 12B). The same results were obtained when 6-OHDA was utilized instead of rotenone (Figure 12B).

Using FAC pretreatment followed by the Parkinson’s inducers, the α-synuclein levels were higher than the rotenone or 6-OHDA alone or dual treatments with FAC and rotenone or 6-OHDA (Figure 12A–C). They suggest that iron overload acts as an activator of α-synuclein mRNA expression. In the case of FAC pretreatment, linalool and geraniol reduced the α-synuclein levels using rotenone or 6-OHDA. However, linalool had a more substantial effect on α-synuclein expression than geraniol (Figure 12C).

## 4. Discussion

There is an emerging demand for using natural compounds as complementary therapies or stand-alone preventive measures [18]. Essential oils from aromatic plants can be extracted from different plant parts like leaves, flowers, or roots [33]. Essential oils contain numerous volatile compounds that can influence each other’s actions. Therefore, even if they have similar compositions, essential oils can have different biological activities [34,35].

In recent years, the effectiveness of essential oils and their components have been investigated in many diseases [20,21,36]. The essential oil components like limonene, α-pinene, or geraniol can regulate apoptosis and affect NFκB, PI3K, and MAPK signal transduction pathways [37]. Others, like β-caryophyllene and α-humulene, modulate STAT3, Akt, and Wnt/β-catenin signaling pathways [38,39].

Linalool, the major component of *Lavandula angustifolia* L., has been revealed to have anti-inflammatory, antimicrobial, and antioxidant properties [29,40,41]. Geraniol of *Pelargonium graveolens* L’Hér. in Aiton acquires antimicrobial, anti-tumor, anti-inflammatory, and antioxidant properties [42,43,44].

The chemical properties of volatile compounds can penetrate the blood–brain barrier and are valuable candidates as neuroprotective agents for distinct neurodegenerative diseases [45,46]. Various essential oils were examined in vitro and in vivo neurodegenerative models like Alzheimer’s and Parkinson’s diseases [30,32] and were proven to alleviate inflammation, oxidative stress, and α-synuclein aggregation [24,25,26,27]. Furthermore, linalool and geraniol may further reduce neuroinflammation and protect neurons, which are critical in managing glaucoma and potentially slowing its progression [4].

The in vitro Parkinson’s models were developed by using rotenone, a complex I inhibitor of the mitochondrial respiratory chain [47], and 6-hydroxydopamine, a potent inhibitor of complex I and IV [48], on all-trans retinoic acid differentiated SH-SY5Y cells [49]. Rotenone uncouples the respiratory chain by inhibiting the electron transport between the iron–sulfur centers of complex I and ubiquinone, triggers oxidative stress and proteasome dysfunction, and inhibits microtubule assembly [50,51]. 6-OHDA generates reactive oxygen species, including hydrogen peroxide induced by MAO activity, and derives hydroxyl radicals acting with the direct inhibition of the mitochondrial respiratory chain. The oxidative stress, together with low ATP levels, leads to cell death [48,52,53].

We focused on the beneficial effects of linalool and geraniol, the major compounds of lavender and geranium essential oils, on oxidative stress, antioxidant capacity, ATP levels, inflammation, and iron metabolism of the rotenone and 6-OHDA-induced in vitro Parkinson’s models. Since iron accumulation appears in Parkinson’s disease, we applied two additional experimental models for iron supplementation. Iron ammonium citrate (FAC) was added with rotenone or 6-OHDA in the first case. In the second experiment, FAC pretreatment was utilized before rotenone or 6-OHDA treatments.

Both linalool and geraniol have been described as antioxidant molecules [41,44]. There was no significant difference between the effects of the two essential oil compounds in the case of rotenone treatments. The linalool and geraniol compounds significantly reduced the ROS levels. In the case of 6-OHDA treatments, linalool provided a similar protective effect on the SH-SY5Y cells, but geraniol showed a more prominent action in decreasing the ROS levels. The two essential oil compounds may act via direct free radical scavengers or modify the cells’ antioxidant capacity by increasing the level of protein antioxidants or small-molecule antioxidants [54,55,56].

Linalool can increase glutathione levels and the activity of glutathione peroxidase, catalase, and superoxide dismutase in cases of UV-mediated DNA damage in PC12 cells [57]. Essential oils and their main components can alter the antioxidant capacities in vitro Parkinson’s and Alzheimer’s models [23].

Linalool and geraniol increased the small-molecule antioxidant capacity after rotenone treatment, equivalent to the action of rasagiline. A minor elevation was revealed in the case of protein antioxidants. In the 6-OHDA treatments, both essential oil components significantly elevated the protein antioxidant capacity. Interestingly, in parallel with these results, geraniol decreased, while linalool did not affect the small-molecule antioxidant capacity. When FAC was added to the cells with rotenone or 6-OHDA, it seemed that neither essential oil could counteract the small-molecule antioxidant capacity, only rasagiline. This may suggest that linalool and geraniol directly erase the reactive oxygen species, which may have also originated from the increasing labile iron pool [58]. However, considering the protein antioxidant capacity, linalool and geraniol significantly elevated it after 6-OHDA treatments. Using FAC pretreatment, the cells have time to restore iron before adding rotenone or 6-OHDA. Interestingly, both essential oil components significantly elevated the small-molecule antioxidant capacity. In contrast with this observation, the protein antioxidant capacity was markedly reduced. Based on these findings, linalool and geraniol act differently on the antioxidant capacities of the cells. Moreover, the type of inducers and/or the iron availability influence the antioxidant action of the essential oil compounds.

Essential oils and their major compounds have anti-inflammatory properties due to their role in regulating signal transduction [23]. Linalool reduces inflammation by downregulating the NFκB, MAPK, and C/EBPβ pathways [29,37,59]. Geraniol also inhibits the NFκB and MAPK pathways [60,61]. These pathways are implicated in the transcriptional regulation of pro-inflammatory cytokines like IL-6, IL-1β, or TNFα [62].

Linalool decreased the IL-6 secretions of the SH-SY5Y cells in all three experimental models, even if rotenone or 6-OHDA were used as Parkinson’s inducer. Its effect was more potent compared to geraniol or rasagiline. Interestingly, geraniol was more effective in reducing IL-1β production than linalool and rasagiline. Considering IL-8, both essential oil compounds were able to lower the cytokine secretions. The measured alterations in cytokine secretions do not show a powerful change, which may be due to the lack of action of microglia, which significantly affects both antioxidant and inflammatory processes in Parkinson’s disease [63]. Moreover, essential oils can reduce the inflammatory function of the microglial cells, making them more beneficial in the treatment of neurodegenerative diseases such as Parkinson’s disease and glaucoma [22,59,64]. Essential oils may serve as a psychosocial complementary therapy, alleviating blindness-associated anxiety (13–30%), depression (11–25%), and sleep disturbances in glaucomatous patients (so-called Flammer’s syndrome) [18].

Fractalkine, expressed by the neurons, is crucial in regulating the microglia [7]. During neurodegeneration, the neurons release high levels of soluble fractalkine, which activates microglial cells and causes neuroinflammation [65]. In our in vitro Parkinson’s models, the soluble fractalkine levels increased, and both linalool and geraniol reduced their concentration in the culture medium, suggesting an inhibiting effect of essential oil compounds on the microglial cells and neuroinflammation.

One of the symptoms of Parkinson’s disease is mitochondrial dysfunction, which is mediated by the overexpression of α-synuclein, oxidative stress, and impairment of the mitochondrial electron transport chain [5]. Moreover, iron accumulation enhances the deterioration of ATP synthesis by triggering mitochondrial ROS production [12].

In our experiments, both inducers activate ROS production in the mitochondria, although their mechanisms of action are divergent. Rotenone treatment led to ATP depletion, which could not be restored by adding linalool or geraniol. Only rasagiline could slightly increase ATP production, possibly inhibiting apoptosis [66]. Interestingly, iron availability impacts ATP levels. The 6-OHDA triggers iron release from ferritin in the cytoplasm [67], increasing the labile iron pool and increasing oxidative stress [68]. 6-OHDA treatments decreased the ATP levels and had a more deteriorated effect on the differentiated SH-SY5Y cells when added with FAC. Geraniol significantly elevated the cells’ ATP levels, comparable to the effect of rasagiline, and improved mitochondrial function. Linalool worsened ATP production, suggesting the different working mechanisms of the essential oil components.

Iron accumulation exacerbates the cellular dysfunction in Parkinson’s disease, leading to ferroptosis, an iron-mediated type of apoptosis, and contributes to the progression of the disease [69]. Iron deposition may occur due to the disruption in iron homeostasis involving uptake, storage, and export [70]. The ferritin heavy chain (FTH) is an antioxidant protein-encoding gene since it can remove free and harmful iron from the cytoplasm [71]. However, it has been described that both rotenone and 6-OHDA lead to iron accumulation [72]. Based on the results, it seems that it depends on the iron availability of the cells. Rotenone increased iron accumulation in the presence of iron. Meanwhile, linalool and geraniol decreased the intracellular iron content of the cells and rasagiline. The main difference between their actions is that rotenone decreased the FTH mRNA level compared to the FAC treatment. At the same time, linalool and geraniol did not cause a significant reduction, suggesting the protective effects of the essential oil components against iron-mediated oxidative stress. In the case of 6-OHDA treatments, an increment in iron content was observed with no significant elevation in the FTH expression. At the same time, both essential oil components significantly raised intracellular iron levels with increasing FTH mRNA levels. Proposing the beneficial action of the essential oil compounds prevents excessive ROS production.

According to the literature, rotenone and 6-OHDA induce iron accumulation by increasing iron uptake through TfR1 and reducing iron release via FP iron exporter [72], which was in line with our results. Interestingly, the essential oil components increased FP expression, suggesting that iron release is not disturbed as in rotenone or 6-OHDA treatments. The two essential oil components acted differently on the TfR1 levels. Geraniol decreased TfR1 expression in the case of rotenone treatments but increased it using 6-OHDA, which is in line with the intracellular iron contents. Linalool mainly increased TfR1 levels when using both inducers.

HO-1 also acts as an antioxidant enzyme; on the other hand, it is responsible for the degradation of heme and the liberation of iron into the labile iron pool. Rotenone and 6-OHDA induced the expression of HO-1, triggering the antioxidant effects of the cells in oxidative stress conditions [73]. Linalool and geraniol significantly elevated HO-1 levels compared to inducers’ treatments, proving the protective effects of the essential oil components against oxidative stress. Moreover, linalool and geraniol were much more effective than rasagiline in elevating HO-1 expression. These results suggest that the examined essential oil components may regulate the Nrf2 transcription factor, which has a pivotal role in the antioxidant protection of neuronal cells [74].

Alpha-synuclein also contributes to ROS production and oxidative stress by binding iron and producing superoxide radicals [75,76]. Based on previous results and the known link between α-synuclein and iron, it has been suggested that essential oils may affect its expression. Both linalool and geraniol successfully lowered the mRNA expression of α-synuclein. Linalool was more effective than geraniol or rasagiline, showing the regulatory relationship between essential oil components and synuclein.

Our study has described the beneficial effects of two major essential oil compounds, linalool and geraniol, on oxidative stress, inflammation, and iron metabolism of rotenone and 6-OHDA-treated differentiated SH-SY5Y cells.

The results revealed that both essential oil compounds reduce oxidative stress by modifying the antioxidant capacity of the differentiated SH-SY5Y cells. They lower the pro-inflammatory cytokine secretion. Linalool decreased IL-6 production, geraniol reduced IL-β secretion, while both essential oil components lowered the IL-8 levels, showing the distinct effects of the examined main components on cytokine regulation. Linalool and geraniol altered the intracellular iron content and FTH expression, suggesting a protective role against iron-mediated ROS production. Moreover, both essential oil compounds elevated the mRNA level of the iron exporter FP, contributing to iron release and HO-1 expression acting as an antioxidant gene. Based on the results, both linalool and geraniol significantly lowered the mRNA expression of α-synuclein, suggesting the role of essential oil compounds in regulating α-synuclein.

In summary, linalool and geraniol are relevant candidates as alternative therapies for Parkinson’s disease to decrease oxidative stress and inflammation. However, this study has some limitations. In the Parkinson’s models, only neuronal cells were used, which should be supplemented with the brain’s immune cells, microglia, which have a crucial role in the development and progression of Parkinson’s disease. Our research group is investigating the effects of essential oils and their major compounds on neuron–microglia co-cultures. Furthermore, it should be mentioned that in vivo studies are needed to reveal the effectiveness, metabolism, and safety issues of linalool and/or geraniol in the prevention or treatment of Parkinson’s disease. Some previously published results [21,22,23] have shown the promising neuroprotective effects of essential oils and their compounds in neurodegenerative diseases because of their anti-inflammatory and antioxidant characteristics as well as their ability to penetrate across the blood–brain barrier.

## Figures and Tables

**Table 1:** Real-time PCR primer list.

Primer	Sequence 5′→3′
α-synuclein forward	TTCTGGAAGATATGCCTGTG
α-synuclein reverse	AGTCTTGATACCCTTCCTCA
FTH forward	GAGGTGGCCGAATCTTCCTTC
FTH reverse	TCAGTGGCCAGTTTGTGCAG
FP forward	AAAGGAGGCTGTTTCCATAG
FP reverse	TTCCTTCTCTACCTTGGTCA
TfR1 forward	CATGTGGAGATGAAACTTGC
TfR1 reverse	TCCCATAGCAGATACTTCCA
HO-1 forward	ACCCATGACACCAAGGACCA
HO-1 reverse	ATGCCTGCATTCACATGGCA
GAPDH forward	TGTTCCAATATGATTCCACCC
GAPDH reverse	CCACTTGATTTTGGAGGGAT

## Data Availability

The original contributions presented in this study are included in the article/Supplementary Materials; further inquiries can be directed to the corresponding author.

## Supplementary Materials

The following supporting information can be downloaded at https://www.mdpi.com/article/10.3390/antiox13080917/s1. Figure S1: Viability measurements of the differentiated SH-SY5Y cells after various treatments.
