# Supplementary material for: Linalool and Geraniol Defend Neurons from Oxidative Stress, Inflammation, and Iron Accumulation in In Vitro Parkinson’s Models
Source: Antioxidants (Basel). 2024 Jul 29;13(8):917. doi: 10.3390/antiox13080917 (PMC11351228; doi:10.3390/antiox13080917)
Supplement: Supplementary file 1 [file antioxidants-13-00917-s001.zip › Supplementary information.pdf]

Linalool and geraniol defend neurons from oxidative stress, inflammation, and iron accumulation in *in vitro* Parkinson's models

Edina Pandur [1], Balázs Major [1,2], Tibor Rák [2,3], Katalin Sipos [1], Adrienne Csutak [2], and Györgyi Horváth [3,*]

[1] Department of Pharmaceutical Biology, Faculty of Pharmacy, University of Pécs, Pécs, Hungary; edina.pandur@aok.pte.hu, majbazsa@gmail.com, katalin.sipos@aok.pte.hu
[2] Department of Ophthalmology, Medical School - Clinical Centre, University of Pécs, Pécs, Hungary; csutak.adrienne@pte.hu
[3] Department of Pharmacognosy, Faculty of Pharmacy, University of Pécs, Pécs, Hungary; rak.tibor@pte.hu, gyorgyi.horvath@gytk.pte.hu
* Correspondence: gyorgyi.horvath@gytk.pte.hu

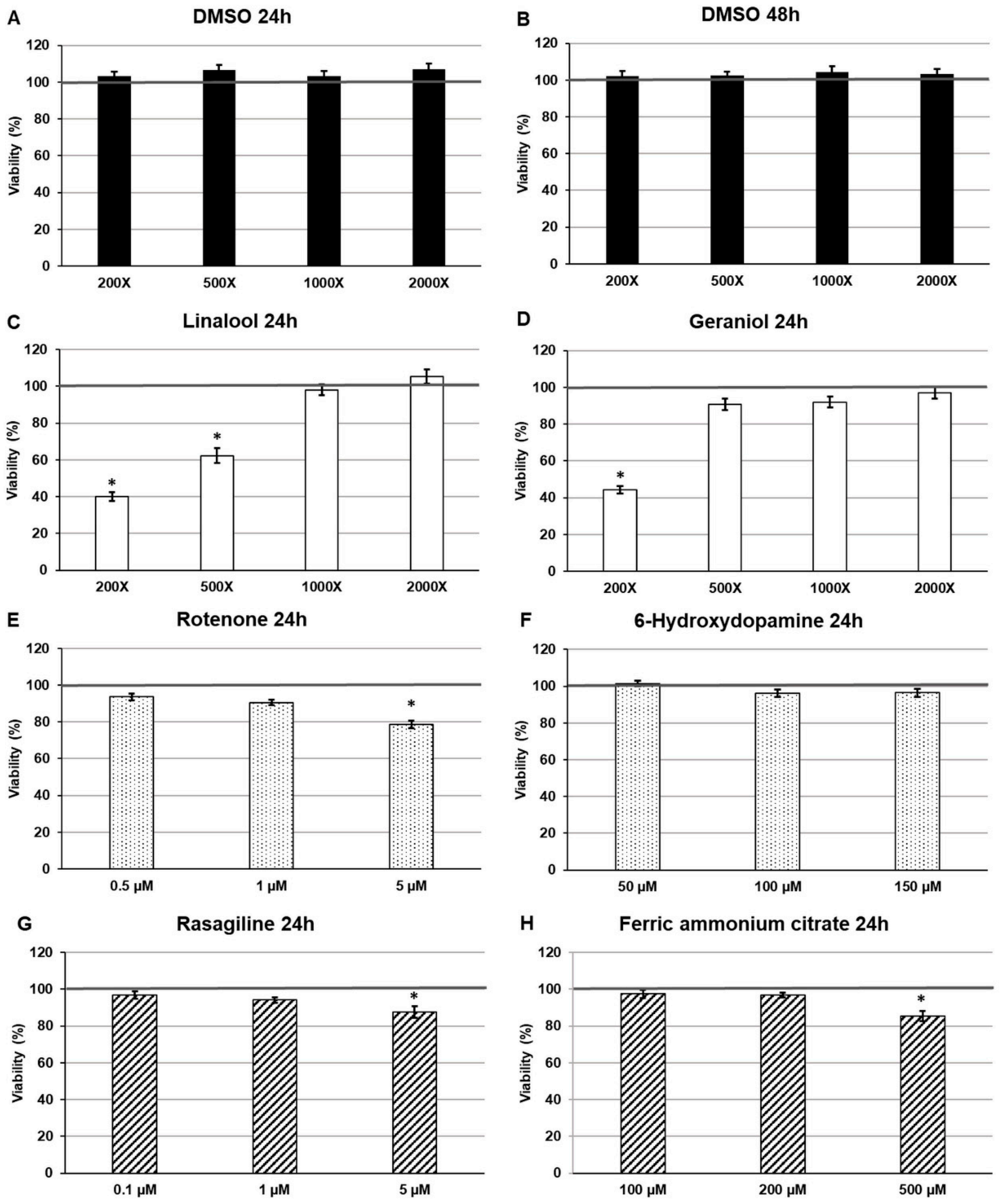

Supplementary figure 1. Viability measurements of the differentiated SH-SY5Y cells after various treatments. Viability was determined using a resazurin-based Toxo-8 kit. DMSO was used as a carrier of the essential oil components and to dilute rotenone. (A-B) The viability of the cells after DMSO treatment compared to the untreated cells (considered as 100%). (C-D) The viability of the cells after linalool and geraniol treatments compared to the DMSO-treated cells. (E) The viability of the rotenone-treated cells compared to the DMSO-treated cells. (F) The viability of the 6-hydroxydopamine-treated cells compared to the untreated control cells. (G) The cells' viability after adding rasagiline compared to the untreated control cells. (H) The viability of the ferric ammonium citrate-treated cells compared to the control cells. The columns represent the mean values ± SD of three independent experiments. The number of technical replicates was 8 per treatment in each experiment. The asterisk shows $p<0.05$ compared to the control.
